# Effect of a subsequent pregnancy on anal sphincter integrity and function after obstetric anal sphincter injury (OASI)

**DOI:** 10.1007/s00192-020-04607-8

**Published:** 2020-12-02

**Authors:** Nicola Adanna Okeahialam, Ranee Thakar, Abdul H. Sultan

**Affiliations:** 1 Croydon Urogynaecology & Pelvic Floor Reconstruction Unit, Croydon University Hospital, Thornton Heath, UK; 2 St George’s, University of London, London, UK

**Keywords:** Obstetric anal sphincter injury, Mode of delivery, Endoanal ultrasound, Anal manometry, Anorectal symptoms

## Abstract

**Introduction and hypothesis:**

Endoanal ultrasound (EAUS) and anal manometry are used in the assessment women with a history of obstetric anal sphincter injury (OASI), both postpartum and in a subsequent pregnancy, to aid counselling regarding mode of delivery (MOD).

**Methods:**

A prospective observational study between 2012 to 2020 was completed. Women were reviewed 3 months postpartum following OASI and in the second half of a subsequent pregnancy. Anorectal symptoms were measured using the validated St Mark’s Incontinence Score (SMIS: asymptomatic to mild symptoms = ≤ 4). Anal manometry (incremental maximum squeeze pressure [iMSP: normal = > 20 mmHg]) and EAUS (abnormal = sphincter defect > 1 h in size) were performed.

**Results:**

One hundred forty-six women were identified and 67.8% had an anal sphincter defect ≤ 1 h in size postnatally. In those with a defect ≤ 1 h, postpartum mean iMSP and SMIS significantly improved in a subsequent pregnancy (*p* = 0.04 and *p* = 0.01, respectively). In women with a defect > 1 h, there was no significant difference between the mean iMSP or SMIS score postnatally compared to a subsequent pregnancy. At both time points, significantly more women had an anal sphincter defect ≤ 1 h and SMIS of ≤ 4 (*p* = 0.001 and *p* < 0.001 respectively) compared to those with a defect < 1 h. In addition, significantly more women had an anal sphincter defect ≤ 1 h and iMSP ≥ 20 mmHg (*p* < 0.001). Overall, out of the 146 women included in this study, 76 (52.1%) with a defect ≤ 1 h also had an iMSP ≥ 20 mmHg and SMIS ≤ 4 at 3 months postpartum.

**Conclusions:**

Women who remain asymptomatic with normal anal manometry and no abnormal sphincter defects on EAUS postnatally do not need to have these investigations repeated in a subsequent pregnancy and can be recommended to have a vaginal delivery. If our protocol was modified, over half of the women in this study could have had their MOD recommendation made in the postnatal period alone.

## Introduction

Obstetric anal sphincter injury (OASI) is a significant risk factor for the development of anal incontinence, with approximately 10% of women developing symptoms within a year following vaginal delivery [1]. However, the pathophysiology of anal incontinence secondary to childbirth is multifactorial and may be due to a number of factors including irritable bowel symptoms, neuropathy and sphincter disruption [2]. Endoanal ultrasound (EAUS) and anal manometry are two modalities that can be used to aid counselling regarding mode of delivery in women with a history of OASI [3, 4]. Three-dimensional EAUS is considered the gold standard investigation to evaluate the structure and integrity of the anal sphincter [3, 5], whilst anal manometry and anorectal symptoms assess anal sphincter function [2]. Persistent anal sphincter defects are detected on EAUS in 34 [6] to 91% [7] of women with a history of OASI, despite primary repair. Compared to women with an intact sphincter, these women are four times more likely to experience anal incontinence symptoms [1].

**Fig. 1:**
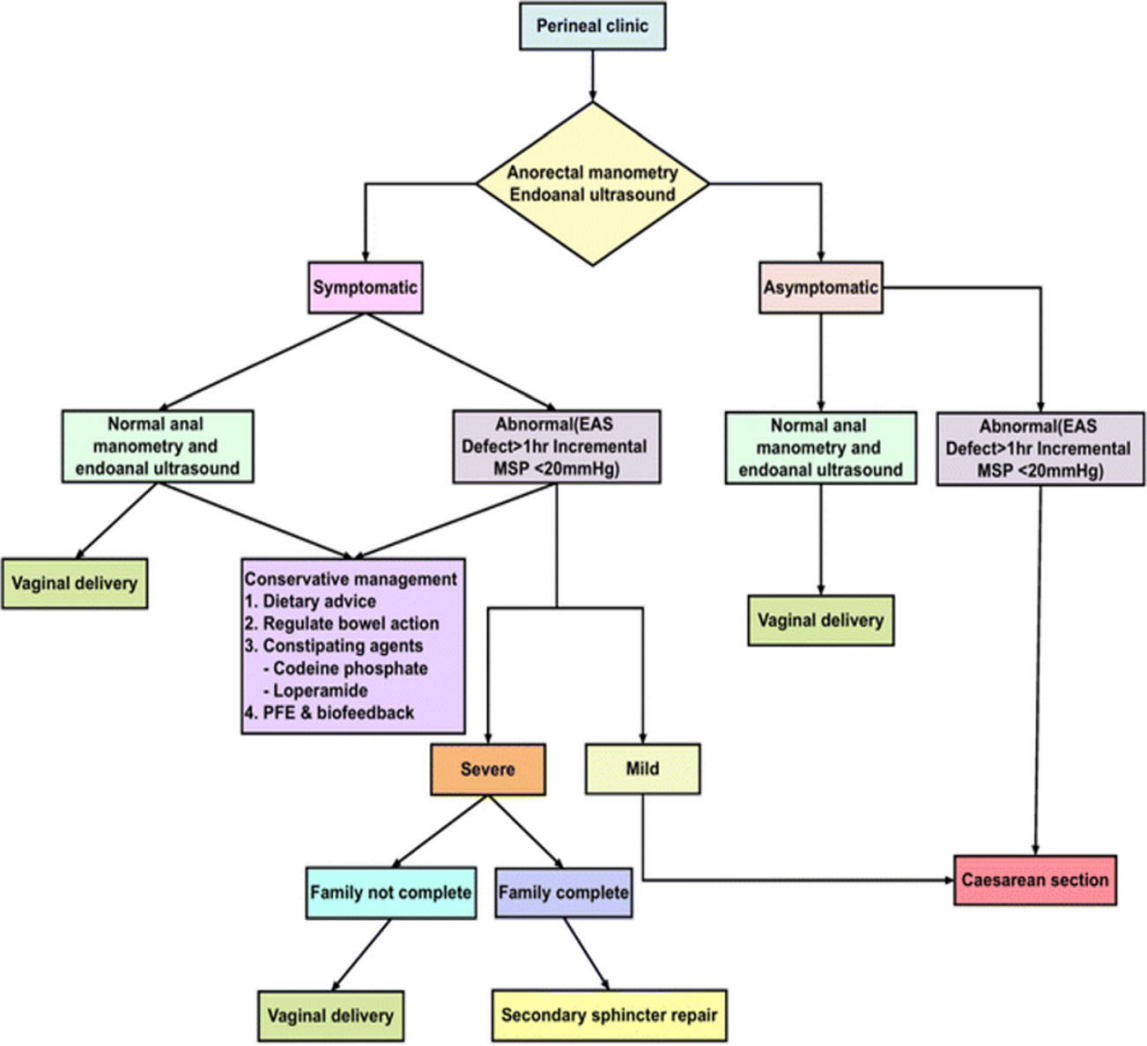
Protocol used in our perineal clinic to aid the recommended mode of delivery decision in a subsequent pregnancy following OASI: [4]

The estimated risk of recurrent OASI in a subsequent vaginal birth is reported in up to 10% of women [8, 9]. The Royal College of Obstetricians & Gynaecologists (RCOG) recommend that if a woman with a history of OASI in a previous pregnancy has anal incontinence symptoms, or has abnormal endoanal ultrasound findings and anal manometry pressures, an elective caesarean section should be considered [10]. A number of centres across the UK and the Republic of Ireland have set protocols using EAUS and anal manometry pressures to enable individualized counselling regarding subsequent mode of delivery for women who have previously sustained OASI [11–13]. Our unit has a dedicated one-stop perineal clinic where all women with a history of OASI have EAUS and anal manometry both at 3 months postpartum and in the second half of a subsequent pregnancy [4, 14]. We follow a protocol for the management of a subsequent delivery following OASI, which has been published previously (Fig. 1) [4].

The aim of this study was to evaluate the need to perform EAUS and anal manometry in both the postnatal and subsequent antenatal period to make recommendations regarding the management of a subsequent pregnancy in women with a previous OASI.

## Materials and methods

Between January 2012 to August 2020, data for all women with a history of OASI who attended the Croydon University Hospital perineal clinic were entered prospectively into the patient database. As this is part of normal practice for the perineal clinic, institutional board and research ethics committee approval was not deemed necessary.

**Fig. 2:**
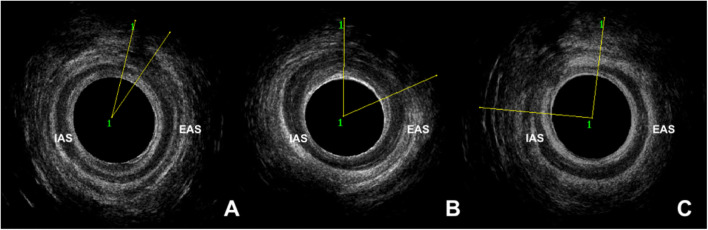
Endoanal ultrasonography findings of three different women 3 months following primary repair OASI. **a** Endoanal ultrasonography showing a scar within the external anal sphincter measuring 20 degrees, equivalent to a defect ≤1 hour (h). **b** Endoanal ultrasonography showing an external anal sphincter defect measuring 63 degrees, equivalent to a defect of approximately 2 h in size. **c** Endoanal ultrasonography showing an internal and external anal sphincter measuring 96 degrees, equivalent to a defect of approximately 3 h in size

Women were reviewed both 3 months postpartum following OASI and antenatally in the second half of a subsequent pregnancy. This included assessment of anorectal symptoms, anal manometry and EAUS findings. Anorectal symptoms were evaluated using the validated St Mark’s Incontinence Score (SMIS), which grades the severity of anal incontinence on a scale of 0 (none) to 24 (severe) [15]. Severity sub-groups included 0–4, 5–8 and > 8 (asymptomatic to mild, moderate and severe) [16]. Anal manometry was performed using a validated Stryker 295–1 Intra-Compartmental Pressure Monitor [17] or the portable Anopress device (*THD* Worldwide, Correggio [RE], Italy) [18]. Maximum resting pressure (normal = 40–103 mmHg) and maximum squeeze pressure (normal =41–121 mmHg) were measured. The difference between these two measurements is the incremental maximum squeeze pressure [iMSP] (normal = > 20 mmHg), which directly correlates with external anal sphincter (EAS) function [3]. Three-dimensional EAUS was performed using the Pro-focus 2202 or Flex-focus 500 ultrasound system (BK Medical, Herlev, Denmark). Anal sphincter defect sizes were measured using a 3-point angle with images taken at the deep (proximal), superficial (mid) and subcutaneous (distal) levels. Images with a defect of ≤ 1 hour (h) (≤ 30° angle) were classified as a scar, a normal finding following primary OASI repair (Fig. 2a) [4, 14]. An anal sphincter defect was defined as abnormal if the defect extended for > 1 h (> 30° angle) (Fig. 2b, c). All images were reviewed independently by one of the two consultants (A.H.S, R.T) experienced in endoanal ultrasound.

Using our protocol, vaginal delivery is recommended in a subsequent pregnancy if a woman is asymptomatic or if her anorectal symptoms are minor, if there is an EAS defect ≤ 1 h and iMSP > 20 mmHg. Caesarean section is recommended to all others [4, 14].

Data were analysed using SPSS version 26.0.0.0. Nominal data are expressed as number and percentage. For continuous data, the mean (SD) was calculated. Continuous variables were compared using Student’s *t*-test, whereas the Fisher’s exact test was used for categorical variables. In addition, the paired *t* test was used for the longitudinal comparison of anal manometry and SMIS at both time points. A corresponding *p* value of <0.05 was considered statistically significant.

## Results

During the study period 146 women were reviewed at two time points: 3 months postpartum and then in the second half of a subsequent pregnancy. The mean time between these two time points was 30 (range 11–78) months. Table 1 describes the patient characteristics and delivery details. Of the 146 women at the postnatal visit, 93.2% (*n* = 136) were primiparous; 54.1% (*n* = 79) had a spontaneous vaginal delivery and 45.9% (*n* = 67) had an instrumental delivery. Ninety-nine (67.8%) women had an anal sphincter defect of ≤ 1 h on EAUS and 47 (32.2%) had a defect > 1 h. Table 1 describes the patient characteristics and the delivery details of these women. Women with a defect > 1 h had a significantly lower BMI (23.6 vs 25.7 kg/m^2^, *p* = 0.01) and higher infant birthweight (3620.8 vs 3430.7 g, *p* = 0.02) compared to those with a defect of ≤ 1 h. There was also a significant difference (*p* = 0.001) between the two groups in the grade of tear diagnosed at delivery. In the women with anal sphincter defect of ≤ 1 h on EAUS, at delivery, 38 (38.4%) were diagnosed with a 3a tear, 48 (48.5%) with a 3b tear, 10 (10.1%) with a 3c tear and none with a fourth-degree tear, whilst in those with a defect > 1 h, 8 (17.0%) were diagnosed with a 3a tear, 29 (61.7%) with a 3b tear, 5 (10.6%) with a 3c tear and 5 (10.6%) with a fourth-degree tear.

**Table 1:** Patient and delivery characteristics

	Defect ≤ 1 h *N* = 99 Mean (SD)/*n* (%)	Defect > 1 h *n* = 47 Mean (SD)/*n* (%)	*p* value
Age (years)	29.1 (4.2)	30.4 (3.9)	0.09*
BMI kg/m^2^	25.7 (4.9)	23.6 (3.2)	0.01*
Parity			
1	91 (91.9)	45 (95.7)	0.50**
≥ 2	8 (8.2)	2 (4.3)	
Ethnicity			
White	46 (46.5)	18 (38.3)	0.70**
Black	9 (9.1)	5 (10.6)	
Asian- Indian, Pakistani, Bangladeshi	32 (32.3)	18 (38.3)	
Asian- Chinese	2 (2.0)	1 (2.1)	
Mixed ethnicity	2 (2.0)	1 (2.1)	
Other	8 (8.1)	3 (6.8)	
Delivery			
SVD	59 (59.6)	20 (42.6)	0.42**
Vacuum	19 (19.2)	11 (23.4)	
Forceps	13 (13.1)	9 (19.1)	
Ventouse + forceps	8 (8.1)	7 (14.9)	
Infant birth weight (g)	3430.7 (423.1)	3620.8 (458.9)	0.02*
Clinical grade of tear			
3rd- not specified	3 (3.0)	0 (0)	0.001**
3a	38 (38.4)	8 (17.0)	
3b	48 (48.5)	29 (61.7)	
3c	10 (10.1)	5 (10.6)	
4th	0 (0)	5 (10.6)	

N = number

SVD = spontaneous vaginal delivery

Bold-Italic = Significant *p*-value (< 0.05)

^*^p value calculated using Student’s *t*-test

^**^p value calculated using Fisher’s exact test

Table 2 describes discrepancies between initial EAUS findings (OASI defect classification) 3 months postpartum and in the subsequent pregnancy. Out of the 146 women, 99 (67.8%) were diagnosed with a defect ≤ 1 h postnatally. Scan discrepancy was found in five (3.4%) patients. All five were due to differences of > 1 h in EAS defect size, meaning that their anal sphincter defect was then re-classified to > 1 h in size. Overall, 97 (66.4%) women had a sphincter defect of ≤ 1 h on EAUS at both time points.

**Table 2:** Discrepancies between OASI defects diagnosed on EAUS at 3 months postnatally and the in the subsequent pregnancy

EAUS findings	Postnatal n(%) (*n* = 146)	Antenatal n(%) (n = 146)	Scan discrepancy n(%)
Defect ≤ 1 h	99 (67.8)	100 (68.5)	5 (3.4)
Isolated EAS defect > 1 h	35 (24.0)	33 (22.6)	6 (4.2)
Isolated IAS defect > 1 h	4 (2.7)	3 (2.1)	1 (0.7)
IAS + EAS defect > 1 h	8 (5.5)	10 (6.8)	2 (1.4)

EAS = external anal sphincter

IAS = internal anal sphincter

In total, 44 (30.1%) women had a sphincter defect > 1 h in size at both time points. Thirty-five (24.0%) women were diagnosed with an isolated EAS defect > 1 h postnatally. Scan discrepancy was found in six (4.2%) patients. Five were due to EAS defects differing by 1 h in size and one had an additional internal anal sphincter (IAS) defect noted in the antenatal period. Four (2.7%) women were diagnosed with an isolated IAS defect > 1 h postnatally. Scan discrepancy was found in one (0.7%) patient with an additional EAS defect noted in the antenatal period. Eight (5.5%) women were diagnosed with an EAS and IAS defect > 1 h postnatally. Scan discrepancy was found in two (1.4%) women, with one EAS defect differing in size by 1 h and one additional IAS defect noted in the antenatal period.

Table 3 shows a longitudinal comparison among EAUS findings, anal manometry and reported anorectal symptoms at 3 months postpartum and in the second half of a subsequent pregnancy. In those women with an anal sphincter defect ≤ 1 h, there was a significant improvement (*p* = 0.04) in mean iMSP measured 3 months postpartum (42.4 mmHg [SD ± 26.2]) compared to the subsequent antenatal period (48.0 mmHg [SD ± 26.1]). Also, there was a significant improvement (*p* = 0.01) in anorectal symptoms, with a mean SMIS score reported at 3 months postpartum of 1.2 (SD ± 2.8) compared to 0.5 (SD ± 1.7) in the subsequent pregnancy. In those women with an anal sphincter defect > 1 h, there was no significant difference in mean iMSP measured 3months postpartum (31.0 mmHg [SD ± 15.0]) compared to the subsequent antenatal period (35.5 mmHg [SD ± 21.3]). In addition, there was no significant difference with a mean SMIS score reported at 3 months postpartum: 2.5 (SD ± 3.7) compared to 2.0 (SD ± 3.4). In total, 63.9% (*n* = 62) and 90.7% (*n* = 88) of women with sphincter defects ≤ 1 h showed an improvement/no change in iMSP and SMIS, respectively, in a subsequent pregnancy. In addition, 61.4% (*n* = 27) and 35 (77.3%) of those with a defect > 1 h showed an improvement/no change in iMSP and SMIS.

**Table 3:** Longitudinal comparison of anal manometry and St Mark’s score 3 months following OASI and in a subsequent pregnancy

	Postnatal Mean (SD)	Antenatal Mean (SD)	*p* value**
Defect ≤ 1 h (*n* = 97)*			
iMSP	42.4 (26.2)	48.0 (26.1)	***0.04***
SMIS	1.2 (2.8)	0.5 (1.7)	***0.01***
Defect > 1 h (*n* = 44)*			
iMSP	31.0 (15.0)	35.5 (21.3)	0.16
SMIS	2.5 (3.7)	2.0 (3.4)	0.51

iMSP = incremental mean squeeze pressure

SMIS = St Mark’s incontinence score

Bold-Italic = Significant *p*-value (< 0.05)

*The five women with scan discrepancies were removed from the analysis

**p value calculated using the paired *t*-test

At both time points, women with a sphincter defect ≤ 1 h had a significantly higher (*p* = 0.002) mean iMSP (postnatal = 42.4 mmHg, subsequent pregnancy = 48.0 mmHg) comparied to those with a defect > 1 h (postnatal = 31.0 mmHg, subsequent pregnancy = 35.5 mmHg). Mean SMIS was also significantly lower in those with a defect ≤ 1 h (postnatal = 1.2, subsequent pregnancy = 0.5) compared to those with a defect >1  h (postnatal = 2.5, subsequent pregnancy = 2.0) (Table 4). With respect to our perineal clinic protocol, at 3 months postpartum, there was a significant difference (*p* < 0.001) in the number of women with an iMSP ≥ 20 mmHg and a sphincter defect > 1 h (*n* = 9 [19.1%]) compared to those with a defect ≤ 1 h (*n* = 85 [85.6%]). Also, there was a significant difference (*p* = 0.001) in the number of women with a SMIS ≤ 4 (minor symptoms) with a sphincter defect > 1 h (*n* = 33 [70.2%]) compared to those with a defect ≤ 1 h (*n* = 88 [88.9%]). In a subsequent pregnancy, there was a significant difference (*p* < 0.001) in the number of women with an iMSP ≥ 20 mmHg and a sphincter defect > 1 h (*n* = 35 [76.1%]) compared to those with a defect ≤ 1 h (*n* = 93 [93.0%]). In addition, there was a significant difference (*p* = 0.003) in the number of women with a sphincter defect > 1 h and a combination of an iMSP ≥ 20 mmHg and SMIS ≤ 4 (*n* = 30 [65.2%]) compared to those with a defect ≤ 1 h (*n* = 87 [87.0%]). Table 4 further compares anal manometry and St Mark’s scores 3 months following OASI and in a subsequent pregnancy in women with or without a defect > 1 h diagnosed on EAUS. Overall, out of the 146 women included in this study, 76 (52.1%) with a defect ≤ 1 h also had an iMSP ≥ 20 mmHg and SMIS ≤ 4 at 3 months postpartum.

**Table 4:** Comparison of anal manometry and St Mark’s scores 3 months following OASI and in a subsequent pregnancy in women with or without a defect > 1 h on endoanal ultrasound (EAUS)

	Postnatal EAUS			Antenatal EAUS		
	Defect > 1 h mean (SD)/n (%) (n = 47)	Defect ≤ 1 h mean (SD)/n (%) (*n* = 99)	*p* value	Defect > 1 h mean (SD)/n (%) (*n* = 46)	Defect ≤ 1 h mean (SD)/n (%) (*n* = 100)	*p* value
Mean iMSP (mmHg)†	31.0 (15.0)	42.4 (26.2)	0.002*	35.5 (21.3)	48.0 (26.1)	0.01*
iMSP ≥ 20 mmHg	9 (19.1)	85 (85.6)	<0.001**	35 (76.1)	93 (93.0)	<0.001**
iMSP < 20 mmHg	38 (80.9)	14 (14.1)	0.001**	11 (23.9)	7 (7.0)	0.481**
Mean SMIS†	2.5 (3.7)	1.2 (2.8)	0.04*	2.0 (3.4)	0.5 (1.7)	0.01*
SMIS ≤ 4	33 (70.2)	88 (88.9)	0.001**	39 (84.8)	97 (97.0)	<0.001**
SMIS > 4	14 (29.8)	11 (11.1)	0.27**	7 (15.2)	3 (3.0)	0.344**
iMSP ≥ 20 mmHg + SMIS ≤4	29 (61.7)	76 (76.8)	0.08**	30 (65.2)	87 (87.0)	0.003**

iMSP = incremental maximum squeeze pressure

SMIS = St Mark’s incontinence score

Bold-Italic = Significant *p*-value (< 0.05)

†The five women with scan discrepancies were removed from this analysis

*p value calculated using Student’s *t*-test

**p value calculated using Fisher’s exact test

## Discussion

This observational study of women with a history of OASI was designed to assess the impact of EAUS and anal manometry on their management in the postnatal and subsequent antenatal period. We found that in a subsequent pregnancy following OASI, there was a significant improvement in anal manometry pressures and reported anorectal symptoms compared to 3 months following OASI. To our knowledge, this is the first study to investigate the variation in outcomes using validated anal incontinence scores, anal manometry and EAUS at these two time points using a set protocol which aids clinicians in recommending the mode of subsequent delivery.

When endoanal ultrasound is completed postpartum following OASI by experienced clinicians, the interobserver agreement has been shown to be good [19, 20]. Starck et al. [19] previously investigated the interobserver agreement in the detection of anal sphincter defects using EAUS in asymptomatic women. In their study there was strong interobserver agreement in the detection of anal sphincter defects on EAUS. However, disagreement about the detection of anal sphincter defects occurred in 9 out of 97 women (9.3%), with the majority being due to disagreement surrounding the extent of an EAS defect (eight proximal partial EAS defects and one combined proximal EAS and IAS defect). It is therefore not surprising that in our study, although all scans were reviewed by one of the two consultants experienced in endoanal ultrasound, there was a discrepancy with the findings at the postpartum and subsequent antenatal period in 9.6% of scans, with most being due to disagreement with the extent of an EAS defect by 1 h, meaning their defect was now classified as > 1 h. This highlights that the size of an anal sphincter defect is unlikely to change with a subsequent pregnancy and most changes in scan findings noted are secondary to systematic interobserver error.

In our study, the average time between postpartum review following OASI and assessment in the subsequent pregnancy was 30 months. Injury to the anal sphincter during vaginal delivery can be mechanical, neuropathic or a combination of both [17] with each factor giving rise to anal incontinence. The pelvic floor musculature is innervated by the sacral nerves (S2-S4) from which the pudendal nerve also arises. The EAS is innervated by the inferior rectal branch of the pudendal nerve [21]. Injury to the pudendal nerve may be secondary to mechanical stretching and/or compression of the nerve by the foetal head, a large for gestational age foetus, prolonged second stage of labour or forceps delivery [21, 22]. However, the neuropraxia from stretch or compression injury usually recovers and muscle reinnervation occurs within 6 months [17, 23, 24]. This could explain why the iMSP, which correlates with anal sphincter function [18], increased significantly between the time period of 30 months following OASI and the subsequent pregnancy in those women with a sphincter defect ≤ 1 h. However, another reason for functional improvement is recovery of muscle strength with pelvic and anal sphincter exercises. In our dedicated perineal service, all women who have sustained an OASI are advised to start pelvic floor muscle training (PFMT) [25]. Information is provided using patient information leaflets and mobile health applications prior to discharge [26]. Women who are unable to contract their muscles effectively are referred to the colorectal nurse specialist for electrical muscle stimulation.

Up to 24% of women experience anal incontinence following OASI and repair by 2 months postpartum [27]. Women should be advised about the benefits of PFMT after OASI in reducing anorectal symptoms [28]. It has been shown that compared to initiation of PFMT 6 to 8 weeks after delivery, PFMT initiated within 4 weeks following OASI results in a significantly greater improvement in anorectal symptoms [28]. In particular, the risk of flatal incontinence and liquid stool incontinence was reduced by 50% and 80%, respectively, when PFMT was initiated early [26]. This may explain why anorectal symptoms had significantly improved during the period up to the subsequent pregnancy following OASI. However, although the mean follow-up time was 30 months, this may still be relatively short term with regard to the development and deterioration of anorectal symptoms. Women with a history of OASI tend to develop anal incontinence in their 50s because of additional factors such as ageing and menopause [29].

A recently published meta-analysis evaluating the risk of anal sphincter defects diagnosed on EAUS following OASI showed that 45% of women who had undergone primary repair of OASI did not have a residual defect [1]. At present, in our perineal clinic [4] we assess women who have sustained OASI both at 3 months postpartum and in the second half of a subsequent pregnancy. However, we showed that up to 77% of women (*n* = 76) with no residual defect on EAUS had normal anal manometry and were either asymptomatic or reported minor anorectal symptoms (SMIS ≤ 4) at 3 months following OASI. The proportion of these women with normal anal manometry and an SMIS ≤ 4 increased to 87% (*n* = 87) in subsequent pregnancy. In accordance with our perineal clinic protocol [4] as there was no EAUS defect (> 1 h), a vaginal delivery would have been recommended both at 3 months postpartum and in subsequent pregnancy. It is however important to note that the anal manometry pressures, a direct indicator of anal sphincter function [2] and reported SMIS, were significantly worse in women with a sphincter defect > 1 h compared to those ≤ 1 h. Our study therefore indicates that our current protocol needs re-appraisal for women who are asymptomatic and have normal anal manometry pressures and EAUS findings 3 months postpartum. Based on this study, if the protocol is modified, 76 (52.1%) women in this study could have had their mode of delivery recommendation made in the postnatal period and therefore these women would not require further review in their subsequent pregnancy. This new policy would avoid unnecessary intrusive investigations being repeated, with attendant financial savings.

The strengths of this study include, first, the use of a standardized protocol [4] and validated tools such as the SMIS [15] to assess anal sphincter function following OASI. Second, the study design comprised prospective collection of the data and independent review of EAUS images by two clinicians experienced in endoanal ultrasound. Third, there was relatively long-term reporting of anorectal symptoms (mean follow-up duration at the time of subsequent pregnancy of 30 months). The limitations include the lack of cost-benefit analysis, which would provide further evidence to support a policy change.

In conclusion, this study showed that following OASI the majority of women do not have a residual anal sphincter defect following a primary repair. Also, there is a significant improvement in reported anorectal symptoms and the incremental anal manometry pressure in a subsequent pregnancy. Therefore, women who remain asymptomatic and have normal anal manometry and no residual sphincter defects on EAUS at postnatal assessment do not need to have these investigations repeated in a subsequent pregnancy and can be recommended to have a vaginal delivery. However, as described previously, those postpartum women who are symptomatic, have compromised anal sphincter function or have anal sphincter defects should be reassessed with these investigations in subsequent pregnancy [4].
